# Smart Vision Transparency: Efficient Ocular Disease Prediction Model Using Explainable Artificial Intelligence

**DOI:** 10.3390/s24206618

**Published:** 2024-10-14

**Authors:** Sagheer Abbas, Adnan Qaisar, Muhammad Sajid Farooq, Muhammad Saleem, Munir Ahmad, Muhammad Adnan Khan

**Affiliations:** 1 Department of Computer Science, Prince Mohammad Bin Fahd University, Dhahran 34754, Saudi Arabia; 2 Department of Computer Science, Lahore Garrison University, Lahore 54000, Pakistan; 3 Department of Cyber Security, NASTP Institute of Information Technology, Lahore 54000, Pakistan; 4 School of Computer Science, Minhaj University Lahore, Lahore 54000, Pakistan; 5 College of Informatics, Korea University, Seoul 02841, Republic of Korea; 6 Department of Computer Science, National College of Business Administration and Economics, Lahore 54000, Pakistan; 7 Department of Software, Faculty of Artificial Intelligence and Software, Gachon University, Seongnam-si 13120, Gyeonggi-do, Republic of Korea

**Keywords:** ocular disease, artificial intelligence (AI), explainable AI (XAI)

## Abstract

The early prediction of ocular disease is certainly an obligatory concern in the domain of ophthalmic medicine. Although modern scientific discoveries have shown the potential to treat eye diseases by using artificial intelligence (AI) and machine learning, explainable AI remains a crucial challenge confronting this area of research. Although some traditional methods put in significant effort, they cannot accurately predict the proper ocular diseases. However, incorporating AI into diagnosing eye diseases in healthcare complicates the situation as the decision-making process of AI demonstrates complexity, which is a significant concern, especially in major sectors like ocular disease prediction. The lack of transparency in the AI models may hinder the confidence and trust of the doctors and the patients, as well as their perception of the AI and its abilities. Accordingly, explainable AI is significant in ensuring trust in the technology, enhancing clinical decision-making ability, and deploying ocular disease detection. This research proposed an efficient transfer learning model for eye disease prediction to transform smart vision potential in the healthcare sector and meet conventional approaches’ challenges while integrating explainable artificial intelligence (XAI). The integration of XAI in the proposed model ensures the transparency of the decision-making process through the comprehensive provision of rationale. This proposed model provides promising results with 95.74% accuracy and explains the transformative potential of XAI in advancing ocular healthcare. This significant milestone underscores the effectiveness of the proposed model in accurately determining various types of ocular disease. It is clearly shown that the proposed model is performing better than the previously published methods.

## 1. Introduction

**Figure 1:**
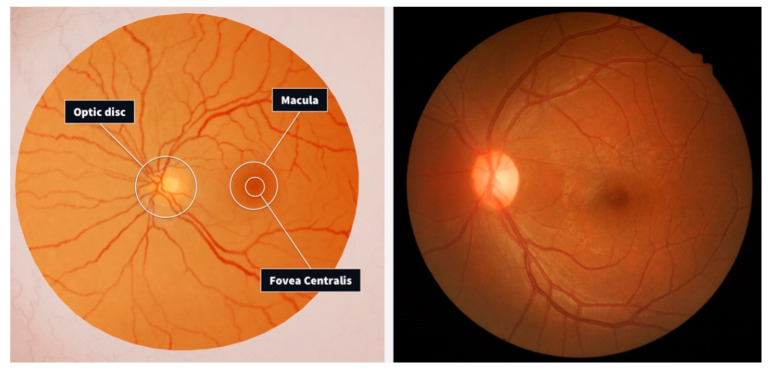
Fundus of the eyeball [2].

In medical eye disease diagnostics, precise predictions and early detection are fundamental tools for efficient treatment and preventing permanent eye damage [1]. Visual diseases like diabetic retinopathy, which is typical for both diabetic patients and the ageing population all over the world, are still one of the most critical global health problems. Artificial intelligence implemented in transfer learning is a great method to speed up and increase the precision of eye disease forecasting [2]. Figure 1 shows the fundus of the eye.

Figure 1 illustrates the fundus of the eyeball, which refers to the posterior aspect where incoming light is focused. This region exhibits several distinctive features within the retina:

- The optic disc, situated centrally, serves as the entry point for sensory nerve fibers and blood vessels via the optic nerve. Despite its vital function, this area is insensitive to light, earning it the moniker “blind spot”;
- Adjacent to the optic disc lies the macula, the central retina, characterized by a slight oval shape. This area houses specialized cone cells responsible for acute vision;
- Within the macula exists a depression known as the fovea centralis, boasting the highest concentration of narrow and elongated cone receptors, optimizing light detection.

Clinicians typically assess various aspects of the fundus, including the optic disc’s shape, color, edges, and cup size, scrutinizing each blood vessel for abnormalities, and examining the macula for signs of degeneration. They specifically target ocular diseases such as diabetic retinopathy, glaucoma, age-related macular degeneration, and cataracts, with a focus on early detection and intervention [3].

In the healthcare industry, which is rapidly advancing, taking artificial intelligence to a prominent role in the diagnostic process is becoming popular among professionals. Radiology and computerized tomography with data and radiography have allowed medical staff to detect diseases sooner and even discover unseen pathologies. Through intelligence analytics, disease outbreaks can be predicted using data analysis and disease forecasting. In contrast, AI has not entirely taken over medical imaging yet; most AI competencies are confined to isolated tasks and still require manual annotation of input data. However, this is an unrealistic goal for providing a desirable smart learning environment and getting acquainted with the complexities of modern medical systems [3].

Today, AI systems utilized in medical imaging are often task-specific and use manually input data as the source of information. This impedes the realization of the ideally aimed state of learning. Nevertheless, there exist some valuable ways to cope with it. An alternative approach is based on deep learning algorithms, mainly based on deep neural networks, which have managed to learn from huge datasets without programming [4]. Another solution is flexible federated learning, which means that multiple collaborators can train a single AI model even on datasets with different sizes and labeling approaches using a single AI model [5]. Furthermore, using the unified representation learning strategies, e.g., REMEDIS, can increase the robustness and the data efficiency of medical imaging AI, resulting in a better data-driven generalization [6,7]. Such developments in AI technologies may serve as tools to remove the obstacles inherent to isolated tasking and manual annotations in medical images.

The implementation of artificial intelligence in medical diagnostics has transformed the standards of the medical field. It is also the reason for the recent development of early detection and diagnosis of diseases. The ocular disease prediction field has made significant progress with artificial intelligence and some applied transfer learning techniques. The implementation of transfer learning in ocular disease prediction showed the possibility of employing a model trained well to identify and categorize ocular images effectively. Transfer learning eludes the need to make specific datasets for every new task by taking knowledge gained from one task and redirecting it to another. Using large datasets and complex patterns, the accuracy of disease prediction increases, and the robustness is improved.

Adopting transfer learning in the development of ocular disease diagnosis endures all shortcomings of traditional methods as it provides the capacity to cope with a dynamic healthcare environment by fitting them into new situations perfectly. It allows high technology leaders to determine the limits of creativity and make accurate diagnoses that result in outcomes that are noticeably better than average technology. The development of transfer learning to act as a “visual” predictor of ocular disease can be insightful for the vision of experts for a prospective successful evolution of ophthalmology studies. Utilizing pre-trained models and large datasets serves the purpose of optimizing the characterization algorithm with more quality and stable results by undoubtedly improving diagnostic prediction accuracy. Initially, it portrays the shortcomings of AI machines that cannot be applied in different cases. Secondly, the continuous learning strategy means that physicians will continually learn throughout their medical careers as technology evolves.

XAI refers to AI systems’ capacity to offer clear and comprehensible explanations for their actions and decisions. The primary objective of XAI is to make these systems’ behavior transparent to humans by clarifying the mechanisms behind their decision-making processes [8]. The field of XAI can improve the quality and effectiveness of decision-making in many areas, such as healthcare, finances, law, and transportation. AI models are usually perceived as ‘black boxes’, which, from the human’s point of view, makes it hard to understand how the machine came to such a decision [9].

XAI techniques have increased AI models’ transparency, reliability, and trustworthiness for diagnosing ocular diseases [10]. They tackle the black-box issue of machine learning and deep learning models by raising interpretability and reliability [11]. XAI has shown a remarkable improvement in the robustness of AI models for ophthalmic applications [12]. Local Interpretable Model-Agnostic Explanations (LIME) have been used to interpret the results from Artificial Neural Networks as a diagnostic tool for glaucoma [13]. Together with image processing techniques, these techniques were deployed to design an interpretable and semi-conscious approach for examining ocular pathologies; this enables the understanding and interpretation of decision-making formulations.

The integration of transfer learning [14] and XAI (known for its human interpretability) leads to an improvement in diagnostic accuracy and sensitivity that can, in turn, translate into better patient outcomes, ultimately leading to increased patient satisfaction. This medical advancement in ocular disease prediction enables transparent ocular disease prediction, bringing more revolutionary changes in the diagnostic fields and, thus, positively contributing to the overall development of healthcare.

## 2. Literature Review

The recent years’ focus on using machine learning to provide efficient prediction of ocular disease has caught a lot of attention in the field. The eye is one of the most significant organs of the body, and early detection and monitoring of its good health is essential.

Various algorithms of machine learning, which are SVM, Random Forest, InceptionV3, MobileNet, Decision Tree, Naive Bayes, AdaBoost, Logistic Regression, k-Nearest Neighbour, Bagging, and Boosting, have been employed to build the best models for disease prediction with the highest accuracy [15]. They consider critical aspects associated with different eye conditions and have produced promising accuracy, precision, sensitivity, and F1-Score [16]. Moreover, the introduction of AI algorithms such as ranker-based feature selection and data splitting methods has consequently augmented the accuracy of these models [17]. AI-based research in retinal biomarkers of systemic diseases has successfully identified age, gender, blood pressure, smoking status, and predicted cardiovascular disorders. It is evident that machine learning and AI-based approaches are practical in accurate ocular disease prediction and will significantly impact clinical practice and future research.

This research [18] addresses the problem of application of machine learning to disease prediction. Three algorithms in the machine-learning processes, Random Forest, Support Vector Machines, and Naive Bayes, were used, and their performances were tested. The highest performance model was a Random Forest one with an average accuracy of 87%. The model was then repeatedly fine-tuned until the accuracy reached a high of 90%. This study brings to light AI’s capability in disease prediction, and the classifier selection and tuning advocated for optimal performance.

In a study [19], researchers focused on the detection of glaucoma disease using machine learning (ML)-based classifiers and a deep learning (DL) model that is based on the Resnet152 convolutional neural network architecture (CNN). The ML classifiers utilized in this project incorporate Support Vector Machine (SVM), K-Nearest Neighbors (KNN), Naive Bayes (NB), Multi-layer perceptron (MLP), Decision Tree (DT), and Random Forest (RF). This method was tested on the ocular disease intelligent recognition dataset. The studies revealed that the two ML classifiers, RF and MLP, had an accuracy of 77% compared to the other ML classifiers. The DL model (CNN: Resnet152) used for the same task and a similar dataset exhibited an even better accuracy of 84%. This research underscores that the best-performing model achieves competitive results compared to specific state-of-the-art approaches. This method lacks external validation on independent data, which can limit the generalizability of results since it is only evaluated with the data it was trained on.

In [20], researchers proposed that a system that utilized the machine learning technique with digital facial images be developed to predict clinical activity score (CAS) in thyroid-associated orbitopathy (TAO). The CAS is a definitive scoring mechanism for detecting the activity of TAO. However, different evaluators’ outcomes may vary, requiring an experienced ophthalmologist to ensure an accurate evaluation. During the study, an AI-assisted system made five assessments of CAS components concerning inflammatory signs in the patients’ facial images; the degree of inflammation was predicted by considering two components of the subjective symptoms. The system achieved satisfactory prediction accuracy for the CES and diagnosis of TAO with sensitivity and specificity variation from 72.7% to 88.1% and 83.2% to 86.9%, respectively. This ML-assisted system may allow us to achieve a uniform and accurate assessment of TAO, which could lead to the early diagnosis and timely treatment of the disease. The Korea Medical Device Development Fund and the Ministry of SMEs and Startups in Korea supported the study.

In the research work [21], a ResNet50 deep learning model was used to train on a fundus image set from the National Taiwan University Hsinchu Branch. This research aimed at detecting and identifying class-related areas for different ganglion cell complex (GCC) thickness conditions, the central focusing field, cropped patches from the fundus, and who operated specific cells. The inclusion of GCC’s thickness led to more precise glaucoma detection. The deep learning algorithms used mainly the Optic Nerve Head (ONH) features for the glaucoma diagnosis that could be found in the clinical guidelines. The models successfully predicted glaucoma cases via a cropped image of the macular area as a single input. With computer-assisted diagnosis, the deep learning models can accurately identify minute morphological changes imaged in the background that might escape clinical detection by experts.

Table 1 summarizes various studies on ocular disease prediction, detailing their methodologies, models, XAI techniques, and limitations. Grampurohit et al. (2020) [18] used a Random Forest model but faced limitations in algorithm exploration and dataset transparency. Badah et al. (2022) [19] employed ResNet 152, lacking external validation for generalizability. Moon et al. (2022) [20] applied machine learning-assisted facial image analysis but required validation on more distinct datasets. Guo et al. (2023) [21] used ResNet50, but a comparison of the model needed an interpretability analysis.

## 3. Proposed Methodology

**Figure 2:**
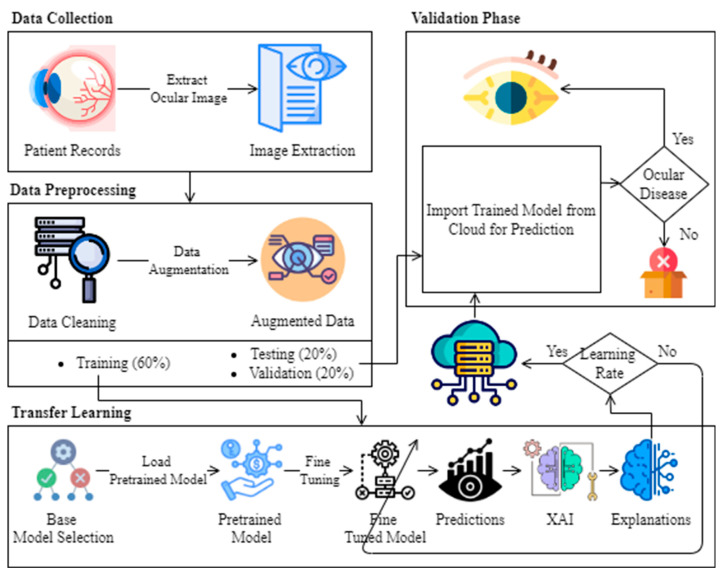
Proposed model for ocular disease prediction using XAI.

In recent years, the health sector has been having increasing problems, especially in the case of eye disease prediction, where precise and transparent decision-making is of the essence. The adoption of AI models in healthcare has raised concern about the complicity of these models, which might undermine trust and understanding among healthcare professionals and their patients. The limitations of traditional black-box AI models and these challenges have been tackled by the proposed model of this research that incorporates explainable artificial intelligence (XAI) approaches to offer insights into the decision-making procedure, resulting in better trust and more accurate ocular disease prediction. This proposed model in Figure 2 aims to consistently increase the authenticity and effectiveness of ocular disease prediction, thus giving good health results and an efficient and reliable healthcare system.

Figure 2 is a comprehensive overview of the proposed model comprising a modified procedure to predict eye disease with the assistance of the XAI method. The primary step involves the data collection of the whole patient record to facilitate image extraction. This process is the key linkage between patient information and consequent analysis stages, and by gathering ocular images of patient information, it is possible to integrate the clinical insights into the predictive model. After collection, the data are transposed to the preprocessing layer responsible for operating properly for eye disease prediction, which, therefore, supports decision-making in the health sector. This layer includes data cleaning and augmentation to prepare the augmented data for transfer learning-based transparent predictions with XAI techniques. The prepared data are later divided into training (60%), validation (20%), and test (20%) datasets.

In the training phase of transfer learning, the training data are used to fine-tune the model’s parameters thereby improving its predictive capabilities. The transfer learning process begins with the utilization of a base model, such as EfficientNet, which is pre-trained on a related task. EfficientNet is particularly suitable for this purpose due to its superior parameter efficiency and accuracy. The model is then fine-tuned on a specific dataset to increase prediction performance, with careful consideration given to its architecture and the efficiency of the base model.

Following fine-tuning, the model’s predictions are processed through the XAI technique, such as Local Interpretable Model-Agnostic Explanations (LIME), to ensure that the outcome is accurate, interpretable, and reproducible by humans. LIME helps to explain individual predictions by approximating the model locally with an interpretable model, making the decision-making process more transparent.

This integration of XAI allows for a comprehensive approach where both high accuracy and the interpretability of results are prioritized, making the model both effective and transparent. This approach underscores the importance of transparency and understanding in medical diagnostics, where explaining the rationale behind AI-driven decisions can significantly enhance trust and reliability. Then, it is further checked whether the learning rate is found or not. If found, the trained explainable patterns are stored on the cloud, whereas in the case of the answer being no, the fine-tuned model is retained, and so on.

Then, in the validation phase, the trained model stored on the cloud is imported into the validation and testing dataset for post-training purposes. The validation data are employed to fine-tune model hyperparameters and assess their performance, ensuring robustness. Subsequently, the testing data are utilized to evaluate the model’s performance objectively, providing a final assessment of its effectiveness before deployment in real-world scenarios. Then, it is checked whether the ocular disease is found or not. If “Yes,” the message is shown that the disease is found, whereas in the case of the answer being no, the process is discarded.

## 4. Simulation Results

This research introduces a transfer learning model enhanced with XAI capabilities to improve transparency in decision-making processes regarding ocular disease prediction. This innovative approach addresses the limitations of traditional methods and demonstrates significant potential in advancing ocular healthcare. The proposed model was applied to a dataset [26], enabling more effective implementation and superior performance outcomes.

**Figure 3:**
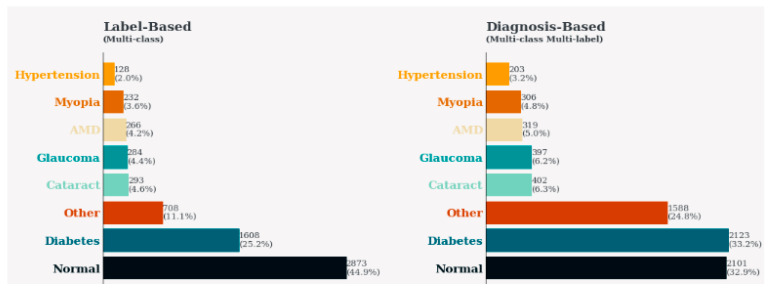
Distribution of classes in ocular disease prediction dataset.

Figure 3 shows an overview of the classes in an ocular disease prediction dataset with projections from label-based and diagnosis-based perspectives. The bar subplot on the left displays the frequency of occurrence for each class as revealed by the labels, placing the corresponding class name on the y-axis. Therefore, the second subplot is aimed at describing the diagnosis basis, where the visualization makes it possible to notice the most frequent ocular diseases. Each plotting of a subplot refers to different classes with their color schemes, and the percentage of occurrences is illustrated on the bars themselves. This visualization allows for quick comparison between class distributions which makes it possible to reveal hidden data composition features and possible biased parts that are to be considered for developing appropriate predictive models in ophthalmology healthcare.

**Figure 4:**
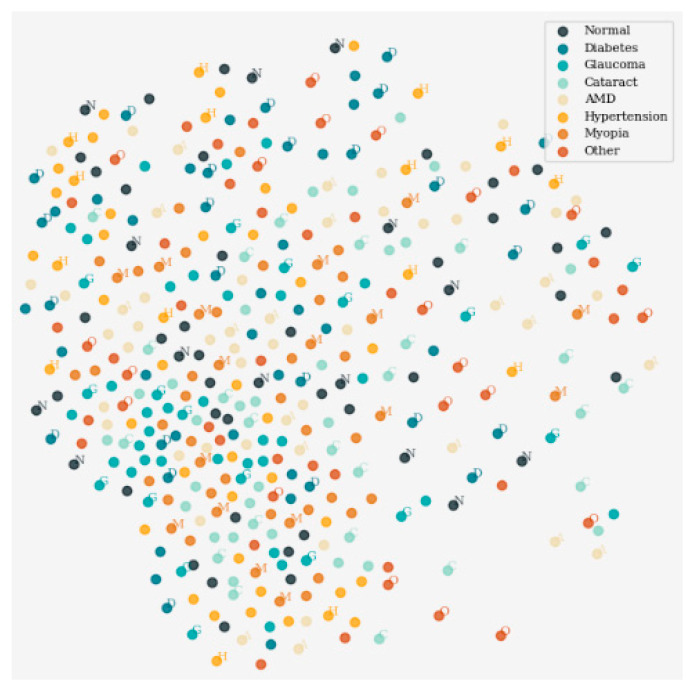
Visualization of dimensionality reduction using t-SNE.

Figure 4 displays the outcomes of dimensionality reduction via the t-distributed Stochastic Neighbor Embedding (t-SNE) approach, which helps in the two-dimensional visualization of data in high dimensions. It demonstrates the distribution pattern of data points on the x–y plane and, therefore, highlights the groups of data points that manifest structure. Every mark is made definitively with different colors to the assigned class markers facilitating inspection and analysis of the underlying data.

**Figure 5:**
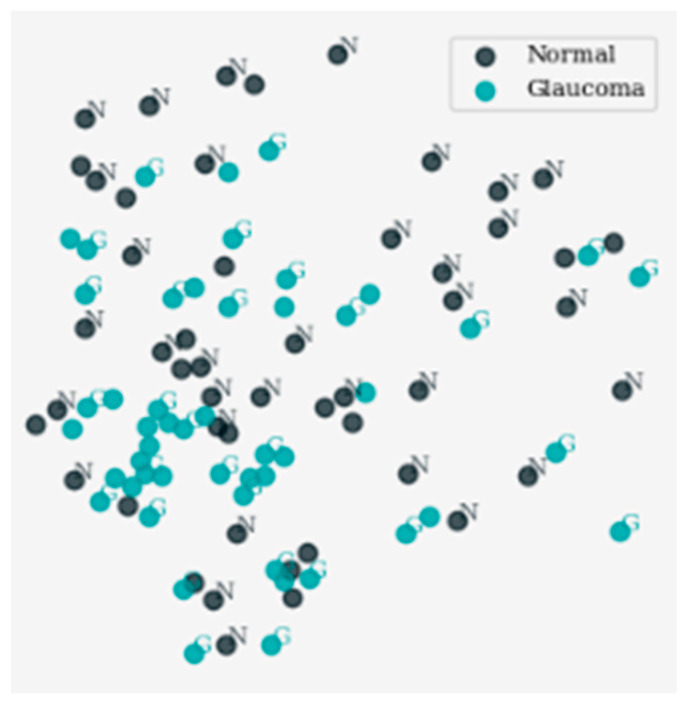
Visualization of dimensionality reduction results for classes ‘N’ and ‘G’ using t-SNE.

Figure 5 displays the area where samples belong to the classes “Normal” and “Glaucoma” with their distribution and clustering trends after the use of t-SNE for dimensionality reduction. This visualization focused only on the activation of these subsets of data giving a 2D reduced view. Each marker has a distinct color which identifies a particular class. This helps in comparing and interpreting the differences between samples that are taken from healthy and glaucoma classes.

**Figure 6:**
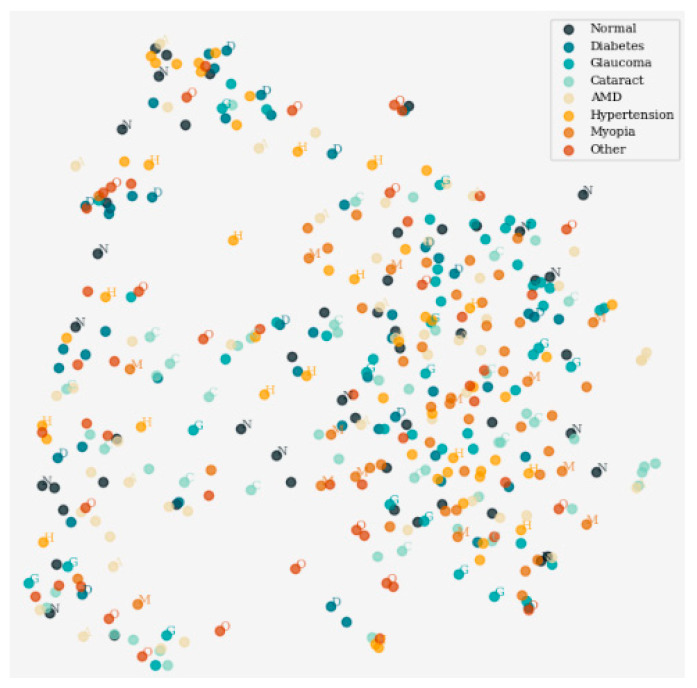
Visualization of dimensionality reduction results using UMAP.

Figure 6 shows the distribution and clustering of data points on a project space of 2D dimensions generated with the Uniform Manifold Approximation and Projection (UMAP) method as the dimensionality reduction. In this representation, each marker, possibly of a distinct color, stands for a separate data point in the class, and it is interpretable by the human eye which can see the relationships between different classes. The figure directs attention to the data structure and stresses the noticeable cluster and prevalent pattern in the reduced space and makes it possible to present the data in this space utilizing UMAP.

**Figure 7:**
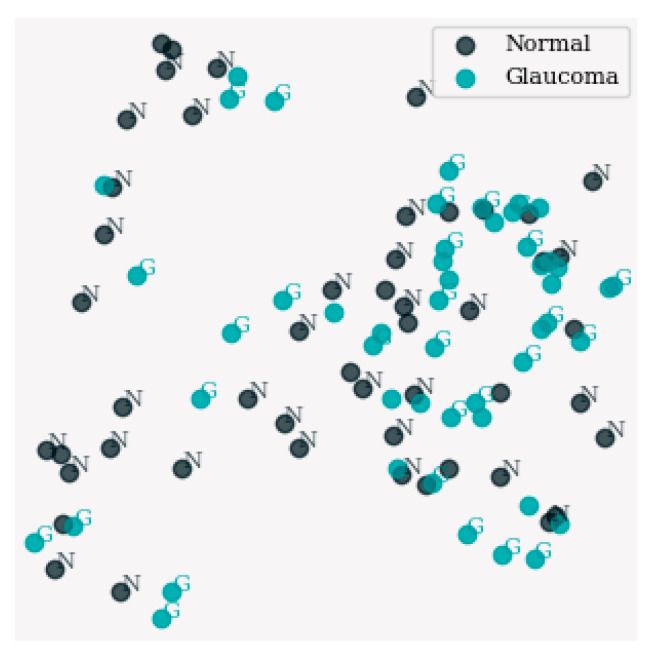
Visualization of dimensionality reduction results for selected samples using UMAP.

Figure 7 shows a subset of results from the dimensionality reduction implemented using UMAP. It displays different clusters and distribution characteristics of selected samples which are highlighted on a low-dimensional graph. There are markers or colors assigned to each data point which are categorized by their class labels and in turn enable visual interpretation of the relations between classes. The figure allows for gaining deeper knowledge of the data structure. It emphasizes the discovery of any visible aggregates or regularities in the subspace, which in turn strengthens the concentration of focus and exploration in UMAP.

**Figure 8:**
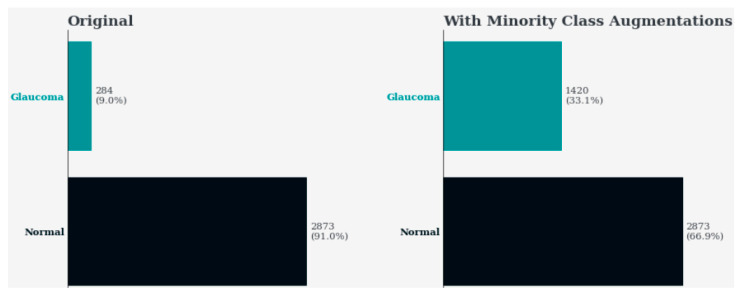
Class distribution before and after minority class augmentations.

Figure 8 displays two subplots side by side, illustrating the distribution of classes in the dataset before and after applying minority class augmentations.

Figure 8 presents two overlapping side-by-side plots that illustrate the diversity of classes in the dataset before and after applying minority class augmentation, respectively. The left subplot (“Original”) shows the percentage distribution of each class in the original dataset. In contrast, the right subplot (“With Minority Class Augmentations”) depicts the adjusted class distribution after augmenting the minority class. It implies the process of augmenting the minority class by increasing the number of samples to balance class distribution. Every class is illustrated by a horizontal bar in both subplots, with the length of the bar representing the percentage of samples from that class. The figure highlights the impact of minority class augmentations on rebalancing the class distribution in the dataset.

**Figure 9:**
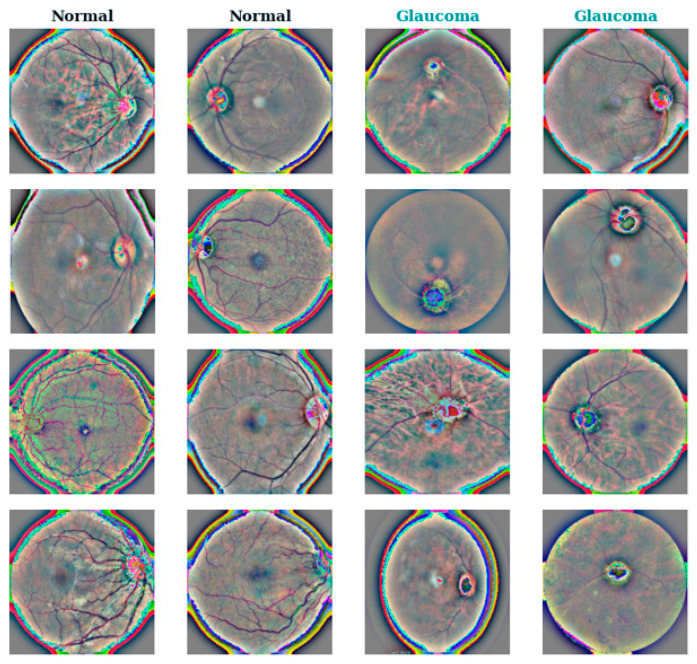
Sample images from training dataset organized by class.

Figure 9 displays a grid of sample images from the training dataset, organized by class. Each row corresponds to a different class, with negative (class 0) and positive (class 1) samples alternating row-wise. The images are randomly selected from the training set for visualization. In each subplot, the title indicates the class to which the displayed samples belong, with colors representing different classes. This visualization provides a visual overview of the characteristics of sample images across classes, aiding in data exploration and analysis during the model development process.

**Figure 10:**
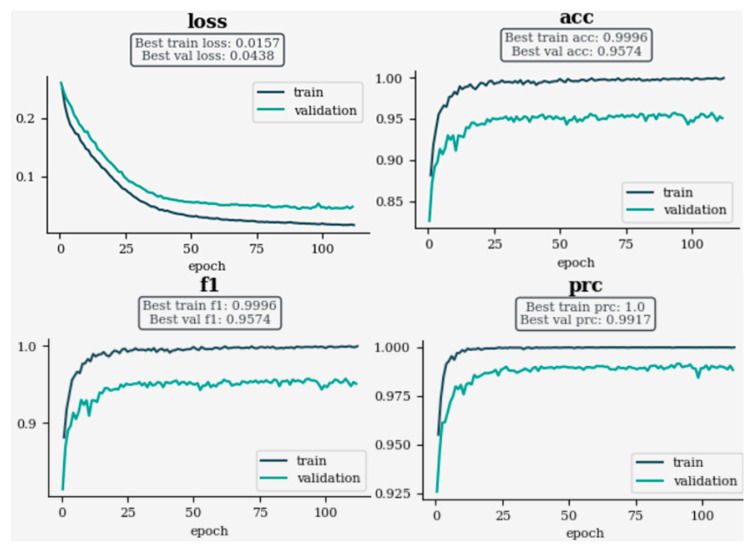
Training history of EfficientNet model for ocular disease prediction with dropout, L1, gamma, and batch parameters.

Figure 10 illustrates the training history of an EfficientNet model for ocular disease prediction, utilizing a dropout rate of 0.1, L1 regularization strength of 0.002, a gamma value of 2, and a batch size of 128. A series of subplots present key performance metrics such as loss, accuracy, F1 score, and precision–recall curve AUC for both the training and validation datasets. Notably, the training loss decreases to 0.0157 while the validation loss reaches 0.0438. Training and validation accuracies are observed to be 0.9996 and 0.9574, respectively. Similarly, F1 scores show high performance, with training and validation values at 0.9996. Precision–recall curve AUC values demonstrate robust model performance, achieving 1.0 for training and 0.9917 for validation. These metrics collectively showcase the model’s learning progress, indicating successful training without overfitting and underscoring the model’s potential for accurate ocular disease prediction.

**Figure 11:**
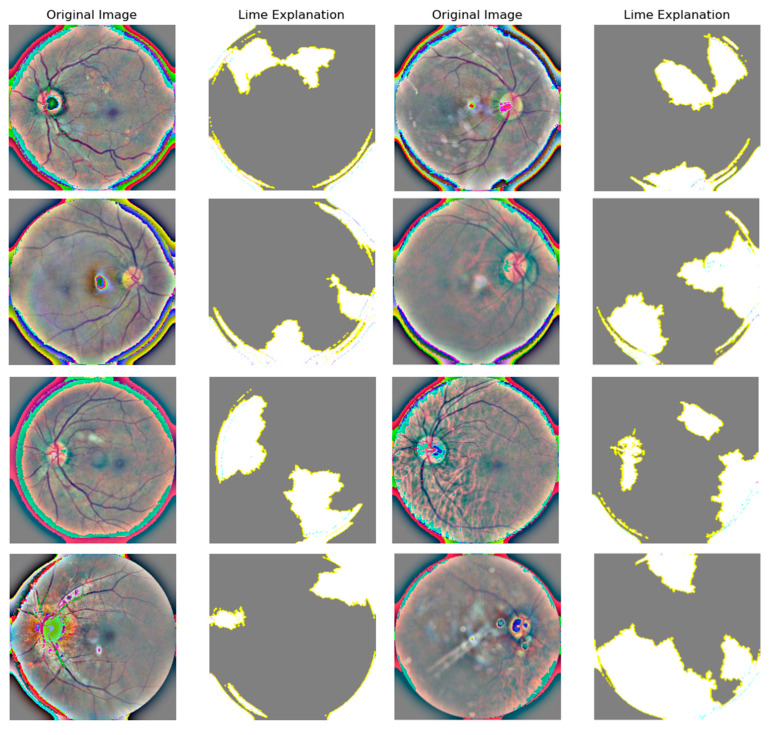
LIME explanations for model predictions.

Figure 11 depicts LIME explanations generated for images used in a transfer learning-based XAI model. LIME explanations aim to elucidate the model’s decision-making process by highlighting the most influential regions within each image. The left side of the figure shows the original image, while the right side presents the LIME explanation, with colored boundaries indicating significant image features. Comparing the original image to the LIME explanation can shed light on the conduct of the model and the regions or parts of the image that mostly influenced the prediction.

This iterative process of generating LIME explanations for multiple images enables a comprehensive analysis of the model’s decision-making across various inputs, fostering transparency and understanding in transfer learning applications.

Table 2 demonstrates a performance comparison of the proposed model using XAI with previous approaches [27,28,29,30,31,32]. It is indicated that the proposed model gives accurate results as compared to previously published methods. The proposed model using transfer learning-based XAI archives 95.74% accuracy and a 4.26% miss-rate.

## 5. Conclusions

The current age poses new challenges in forecasting ocular disease because of variations in the visual symptoms and lack of labeled data that could improve training predictive models. As a result, the traditional methods cannot deal with such complications effectively. Transfer learning involves leveraging knowledge from one task to enhance performance on another, allowing models trained on extensive datasets from related medical domains to be fine-tuned for ocular disease prediction despite limited ocular data availability. Explainable artificial intelligence (XAI) is an emerging approach that aims to make models transparent by providing understandable explanations for their decisions, aiding clinicians in interpreting complex medical data, and fostering trust in AI systems. Transfer learning and XAI address challenges in ocular disease prediction by enabling models to utilize broader datasets and offering interpretable insights into predictions, thereby enhancing diagnostic accuracy and patient care. This research proposed an efficient transfer learning-based XAI model that enables transparent insights into ocular disease prediction. The proposed model utilized in this work achieves an accuracy rate of 95.74%, indicating its efficiency in enhancing diagnostic accuracy and building trust in AI-driven healthcare systems. It is clearly shown that the proposed model is performing better than the previously published methods [27,28,29,30,31,32]. The proposed model, despite its high accuracy, has some challenges such as lack of real-time application and reliance on a specific dataset, necessitating future exploration of real-time data integration and diverse datasets to enhance its robustness and applicability.

## Figures and Tables

**Table 1:** Comparative analysis of previously published approaches.

Research Literature	Methodology	Preprocessing Layer	Predictive Model	XAI Techniques	Limitations
Grampurohit, S. et al., 2020 [18]	Random Forest	×	✔	×	Limited algorithms explored and reliance on tuning, with unclear dataset details.
Badah, N. et al., 2022 [19]	CNN: ResNet 152	×	✔	×	Lacking external validation on independent datasets, potentially limiting generalizability.
Moon, J.H. et al., 2022 [20]	ML-assisted digital facial image analysis	×	×	×	Requires validation on more extensive and diverse datasets for robustness and generalizability.
Guo, J.M. et al., 2023 [21]	ResNet50	×	✔	×	Reliance on a specific dataset without comparing different network architectures, focusing solely on Optic Nerve Head (ONH) diagnosis without interpretability analysis or comparison to human experts or existing methods.
Islam, M.T. et al., 2019 [22]	CNN	✔	✔	×	Lacking transparency and performance.
Lam, C. et al., 2018 [23]	GoogLeNet, AlexNet, and ImageNet	×	✔	×	Lacking transparency and performance.
Fu, H., Cheng et al., 2018 [24]	Ensemble of 4 CNNs	✔	✔	×	Lacking transparency and performance.
Fu, H., Cheng et al., 2018 [25]	DeepCDR	✔	✔	×	Lacking transparency and performance.

**Table 2:** Comparative analysis of the proposed model as compared to the previously published approaches.

References	Models	Accuracy (%)	Miss-Rate (%)
Wang et al., 2020 [27]	EfficientNet	73	27
Lin et al., 2021 [28]	Graph Conv. Network	78.16	21.84
Islam et al., 2019 [29]	Shallow CNN	80.5	19.5
Gour and Khanna et al., 2020 [30]	Two Input VGG16	84.93	15.07
Ning Li et al., 2021 [31]	Inception-v4	88	12
Ou et al., 2022 [32]	ResNet-50	90.3	9.7
Li et al., 2020 [32]	ResNet-101	93	7
Islam, M.T. et al., 2019 [22]	CNN	87.6	12.4
Lam, C. et al., 2018 [23]	GoogLeNet, AlexNet, and ImageNet	74.5, 68.8, and 57.2	25.5, 31.2, and 42.8
Fu, H., Cheng et al., 2018 [24]	Ensemble of 4 CNNs	84.28 (SCES) and 74.95 (SINDI)	15.72 (SCES) and 25.05 (SINDI)
Fu, H., Cheng et al., 2018 [25]	DeepCDR	81.57 (SCES) and 75.85 (SINDI)	18.43 (SCES) and 24.15 (SINDI)
Proposed model using XAI	Transfer Learning	95.74	4.26

## Data Availability

The simulation files/data used to support the findings of this study are available from the corresponding authors upon request.
